# Divergence of stable isotopes in tap water across China

**DOI:** 10.1038/srep43653

**Published:** 2017-03-02

**Authors:** Sihan Zhao, Hongchang Hu, Fuqiang Tian, Qiang Tie, Lixin Wang, Yaling Liu, Chunxiang Shi

**Affiliations:** 1 Department of Hydraulic Engineering, State Key Laboratory of Hydroscience and Engineering, Tsinghua University, Beijing 100084, P. R. China; 2 Indiana University-Purdue University Indianapolis, 723 W Michigan St, SL 118M, IN 46202, USA; 3 Pacific Northwest National Laboratory, Joint Global Change Research Institute 5825 University Research Ct, MD 20740, USA; 4 National Meteorological Information Center, Beijing 10081, P. R. China

## Abstract

Stable isotopes in water (e.g., δ^2^H and δ^18^O) are important indicators of hydrological and ecological patterns and processes. Tap water can reflect integrated features of regional hydrological processes and human activities. China is a large country with significant meteorological and geographical variations. This report presents the first national-scale survey of Stable Isotopes in Tap Water (SITW) across China. 780 tap water samples have been collected from 95 cities across China from December 2014 to December 2015. (1) Results yielded the Tap Water Line in China is δ^2^H = 7.72 δ^18^O + 6.57 (r^2^ = 0.95). (2) SITW spatial distribution presents typical “continental effect”. (3) SITW seasonal variations indicate clearly regional patterns but no trends at the national level. (4) SITW can be correlated in some parts with geographic or meteorological factors. This work presents the first SITW map in China, which sets up a benchmark for further stable isotopes research across China. This is a critical step toward monitoring and investigating water resources in climate-sensitive regions, so the human-hydrological system. These findings could be used in the future to establish water management strategies at a national or regional scale.

Stable isotopes in water (e.g., δ^2^H and δ^18^O) are important indicators of hydrological and ecological patterns and processes^1^. Stable isotopic composition in environmental waters changes as a result of fractionation driven by multiple hydrological and ecological processes. With this unique characteristic, water isotopes have been frequently used to trace atmospheric moisture source^2^,^3^, identify source of groundwater^4^,^5^,^6^ and surface water recharge^7^,^8^, partition evapotranspiration^9^,^10^, and reconstruct paleoclimate^11^.

Stable isotopes have been widely used in geoscience, today, the increasing interest of researchers is focused on addressing issues at national, continental or global scales rather than local^12^. Isoscapes, or mapping large scale spatiotemporal distributions of stable isotope compositions in various environments^13^, provide a framework for large scale fundamental and applied research in a wide range of fields^14^,^15^.

The Global Network of Isotopes in Precipitation (GNIP), established in 1961 by International Atomic Energy Agency (IAEA), is the largest database constituted for monitoring isotopic compositions of precipitation. GNIP has contributed to many studies related to water cycle and climate in different regions all around the world. Additional work on other types of water sources (river, groundwater, etc.) has been frequently conducted at national scale. Kendall and Coplen^16^ provided detailed distribution map of δ^2^H and δ^18^O in US rivers. They showed river water isotopes can act as a proxy for modern precipitation. Katsuyama *et al*.^14^ also analyzed spatial distribution of δ^18^O in stream waters of Japan. Groundwater isoscape was mapped in Mexico^17^ and South Africa^1^, and compared to precipitation.

Natural or artificial mixing of different waters from various origins will propagate the isotopic “signatures” of water source^18^. As a mixture of locally available freshwater (including rivers, lakes, wells and springs), tap water likely reflects integrated features of regional hydrological processes and human activities. Tap water sampling on large scales is more easily achieved than other environmental sources, such as precipitation, groundwater and rivers. Although the isotopic information provided by tap water is not as straightforward as other environmental waters, analyzing tap water isotopic compositions would still provide information on isotopic signals of initial water sources and transport. Bowen *et al*.^18^ presented the first national isoscape map of tap water in US. They found the large extended isotope sampling network can be a useful tool to identify and characterize regional water resource issues within complex human-hydrological system.

China is a large country with significant meteorological and geographical variations, representative of Eastern Asian Monsoon Region. Previous studies on stable water isotopes in the country have mainly focused on precipitation isotopes analysis^19^, moisture tracing on regional scale^20^,^21^ and paleoclimate reconstructions based on GNIP stations^22^.

Built from the six GNIP stations in China, Chinese Network of Isotopes in Precipitation (CHINP), which consists of 29 stations, was established in 2004^23^, which provides basis for analyzing meteorological factors influencing isotope distributions and modelling isotopic composition^19^. In addition to precipitation isotopes research, analysis of deuterium and oxygen-18 in thermal groundwater was conducted in 2008^24^. Based on 90 samples across China, the research discussed the origin of thermal groundwater of different types.

The former studies reveal relations between natural water and environmental factors without taking human-hydrological system into consideration. This study established the first nation-wide network of tap water isotopes in China. The purpose is to set the basis for isotope studies in China and demonstrate the capabilities of network-based isotopic composition data in improving understanding of climate-sensitive, regional water resources. This work may cover the shortage of current data and constitutes a critical step toward monitoring and investigating water consumption system across China. In fine, these findings could be used in the future to establish water management strategies

## Data and Methodology

### Tap water sample acquisition

**Figure 1:**
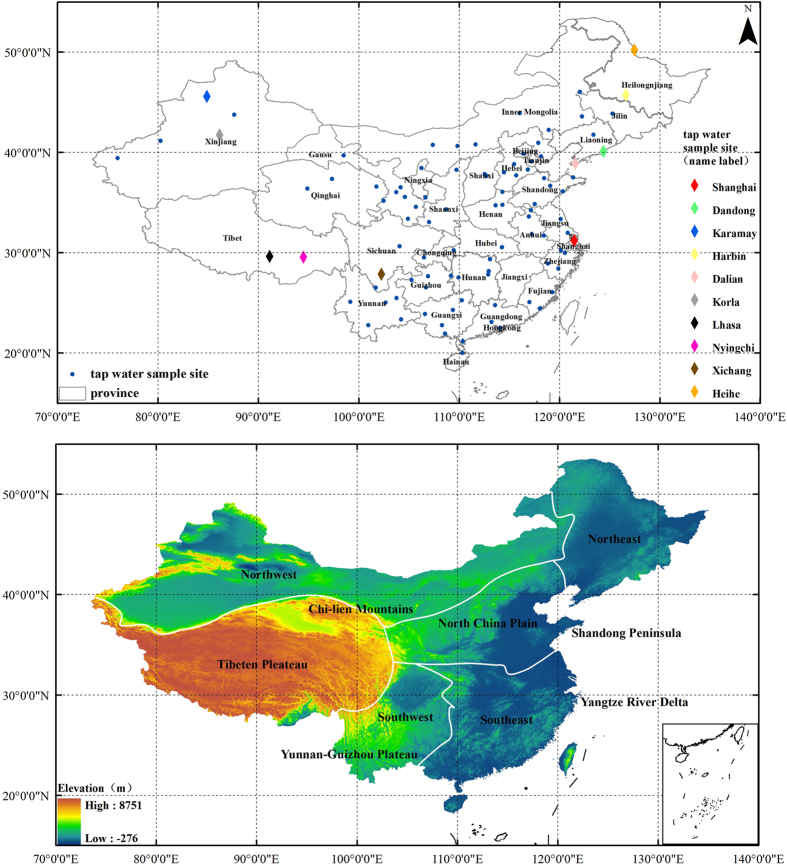
Location and elevation (meters above sea level) of tap water sample locations in China, sampling locations with names mentioned in context are presented as rhombuses in different colors and other general sample locations are presented as circles in royal blue. The elevation map here is presented to give an overview of the China landscape and surrounding environment of the sampling locations. (All of the items were generated with Arcgis 9.3, https://www.arcgis.com/features/index.html).

Characterization of tap water isotope ratio has been realized from December 2014 by nation-wide data collection network representative of spatiotemporal distribution and diversity. Volunteers across China were recruited to collect tap water samples in their living places, from large cities to small rural counties. Volunteers were finally identified for a total number of 95 locations in 32 provinces of China (Fig. 1). This sampling campaign lasted from December 2014 to December 2015. Every month, each volunteer received a returnable plastic box containing one 100 ml plastic bottle with narrow-mouth and an information sheet with instruction. Volunteers were instructed to collect tap water from one tap (home or office) after 5 s of water running^25^. The sampling bottle was filled for approximately four fifths volume in case of breakage caused by the possible freezing during transport. Also the cap was screwed tightly to prevent leakage and eliminate evaporation. Volunteers were asked to record sampling date on a log sheet and indicate whether the water supply is from surface water (including rivers, lakes and reservoirs), groundwater or mixed source. If unknown, detailed information about local drinking water supply system was investigated through internet and expert consultation. All samples were returned to lab in the firm plastic box by express delivery. Tap water samples were prepared, sealed and stored in a cool and dark place a few weeks before analyze. By December 2015, 64 of 95 sampling locations managed to return data for more than 7 months during the 13-month period. A total number of 780 tap water samples have been collected and analyzed for isotopic composition. Table 1 lists location and general climate information of all the sampling locations.

### Isotope analysis and meteorological data

δ^18^O and δ^2^H values of collected samples were analyzed by the Hydrology Laboratory in Tsinghua University. A wavelength-scanned cavity ring-down spectroscopy (WS-CRDS, Picarro L2130i)^1^ was used to analyze all the samples. The measurement precision (standard deviation) is ±0.1‰ and ±1‰ for δ^18^O and δ^2^H, respectively. The isotope values of tap water are reported as per mil (‰) unit relative to the Vienna Standard Mean Ocean Water or VSMOW^26^,


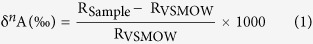


where n is the atomic mass of the heavy isotope of element A, *R*_Sample_ the ratio of heavy to light isotope (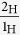 or 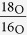) in a sample, and R_VSMOW_ the ratio of heavy to light isotope in international isotopic measurement standard Vienna Standard Mean Ocean Water.

To ensure the accuracy of isotope analysis, each vial was analyzed 6 times. The first three results were abandoned to eliminate memory influence of former sample^27^. During one analysis of a batch of sample vials, the first and last four vials constitutes the standard (Vienna Standard Mean Ocean Water). Regression analysis was conducted to check whether the samples in measure process were problematic^28^. As expected, no samples were identified as problematic.

In order to examine the relationships between tap water isotope and meteorological factors, meteorological data - including the precipitation amount (P, mm), temperature (T, °C), relative humidity (RH, %) and air pressure (PR, kpa) - were collected at observation station in the same city of each sampling location. All the meteorological data were collected from the China Meteorological Data System (http://data.cma.cn/).

## Results and Discussion

### Spatial pattern of tap water isotopes

**Figure 2:**
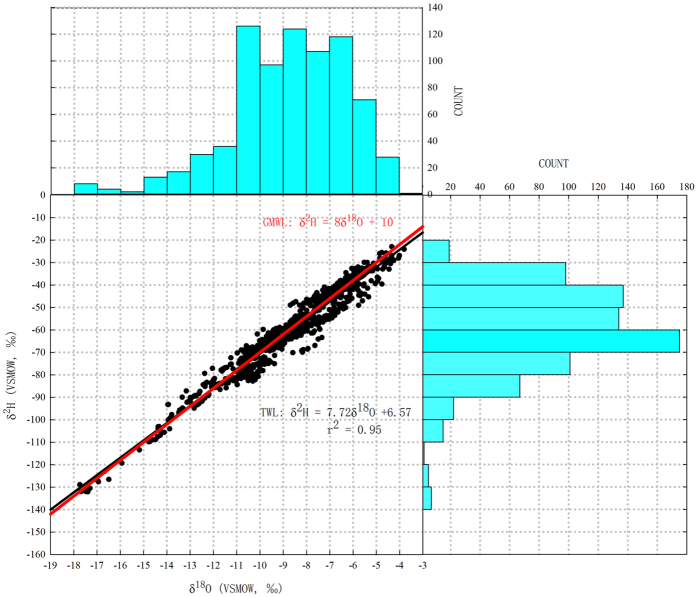
Relationship between δ^18^O and δ^2^H values and their frequency distributions in tap water. The black line represents Tap Water Line (n = 780) and the red line represents the Global Meteoric Water Line.

There was a large range in δ^18^O and δ^2^H values in tap water samples across China. For δ^18^O, the values varied from −17.74‰ to −3.8‰ with an average of −8.75‰. For δ^2^H, the values varied from −132.09‰ to −22.98‰ with an average of −60.92‰. Deuterium excess (calculated as d-excess _tap_ = δ^2^H_tap_ − 8δ^18^O_tap_)^29^ ranged from −5.86‰ to 20.6‰ with an average of 9.3‰. The Tap Water Line (TWL) of China based on the 780 tap water analyses was: δ^2^H = 7.72δ^18^O + 6.57 (r^2^ = 0.95) (Fig. 2). The tap water data clustered near Global Meteoric Water Line (GMWL: δ^2^H = 8δ^18^O + 10)^30^. Both slope and interception in the equation were lower than those in GMWL, which may reflect the effects of evaporation in tap water sources^31^. Compared with Chinese Precipitation Meteoric Water Line^23^, δ^2^H = 7.48δ^18^O + 1.01, TWL exhibited different intercept at 6.57. Although both tap and precipitation datasets were collected across China, the dataset we presented was collected in sequential months from 2014 to 2015. The precipitation data presented in previous study was collected in 29 stations from 2005 to 2010 (no data from 2008). The linear relationship of δ^2^H and δ^18^O in the previous study in the USA^25^ collected from 349 tap water samples is: δ^2^H_August_ = 8.02δ^18^O_August_ + 8.21, δ^2^H_February_ = 8.12 δ^18^O _February_ + 9.49. Compared with GWML, the slope of their dataset is quite similar while the interception is a bit lower. Obviously, there is significant difference in tap water isotopic composition between China and USA as a result of different water supply sources.

**Figure 3:**
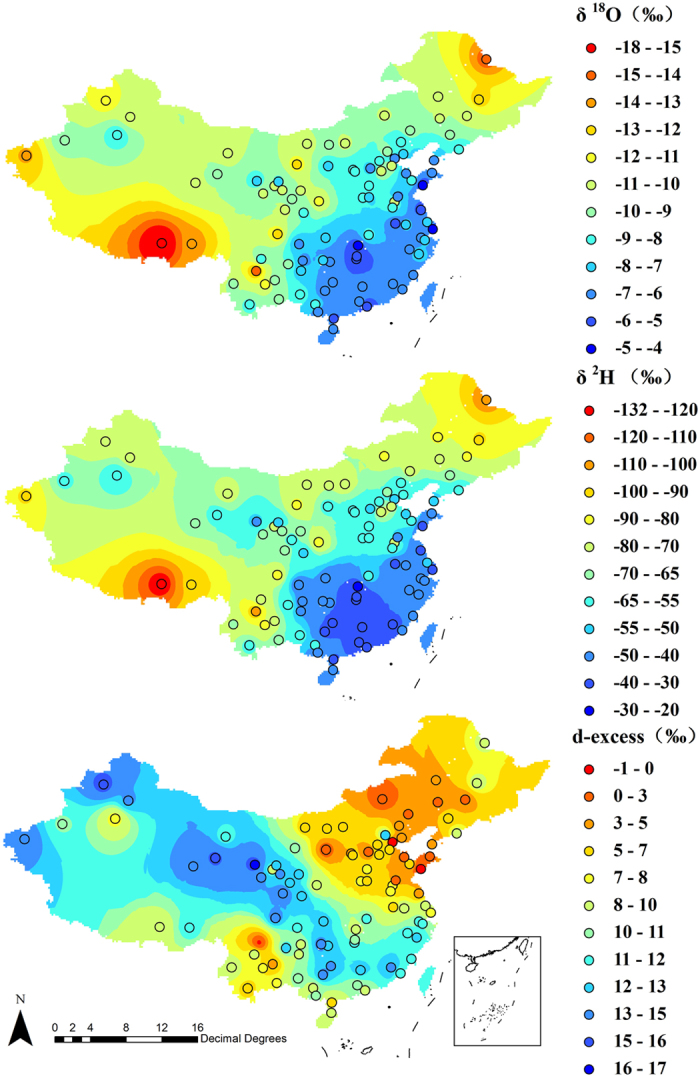
Mean annual observed δ^18^O, δ^2^H and d-excess values in tap water overlaid on a background generated from Inversed Distance Weighted (IDW) interpolation model in ArcGIS 9.3 (https://www.arcgis.com/features/index.html).

Spatial patterns in the isotope values were analyzed using Moran’s test^32^. Moran’s **I** for δ^2^H and δ^18^O were 0.3 and 0.4, Z = 8.08 and 7.1 respectively, p < 0.01 for both, which means the spatial distribution of tap water isotopes is not random. Figure 3 shows a geospatial interpolation mapping of mean annual δ^18^O, δ^2^H and d-excess in contiguous China. Individual tap water’s annual average values are presented on a background colored using Inverse Distance Weighted interpolation model (IDW) in ArcGIS 9.3 (https://www.arcgis.com/features/index.html). In general, tap water isotope values decrease from coastal regions with low latitude and low elevation to inland regions with high latitude and high elevation. This spatial pattern, decrease of isotope values from coastal to inland areas (“continental effect”^33^) is analogous to results in the previous study in the USA^18^.

The highest δ^18^O and δ^2^H values in annual average (−4.75‰ and −30.69‰) appeared in Shanghai on Yangtze River Delta. Other samples with relatively high values were mainly obtained from coastal area in southeastern China (mainly refers to Fujian and Zhejiang province). The location with the lowest values (−17.26‰ for δ^18^O and −129.47‰ for δ^2^H) is Lhasa on Tibet Plateau. Samples obtained from northeastern China (Harbin and Heihe in Heilongjiang province) also presented extremely low values (−12.72‰ and −14.64‰ for δ^18^O, −92.56‰ and −108.68‰ for δ^2^H). All the mentioned sample locations with extreme isotope ratios are highlighted in different colors in Fig. 1.

The extremely low isotope values occurring in these locations could be due to several factors. First, high altitude can lead to extremely low isotopes in precipitation as there is a strong negative correlation between them^34^. Tap water derived from local source that was initially contributed by local precipitation will probably display similar isotope composition at very low ratios. This may, to some extent, explain the extremely low isotope ratios of tap water in Lhasa and Nyingchi (3657 m and 3300 m). Second, in regions with high latitude, e.g., Harbin and Heihe (44.1°N and 50.2°N), isotope ratios in precipitation is strongly linked to local temperatures in high latitudes^35^,^36^. Tap water derived from regions with high latitude and low temperature tends to have lower isotope values. In both regions mentioned above, high latitude and altitude are related to low temperature which can influence isotope fractionation in precipitation.

In contrast with δ^18^O and δ^2^H, deuterium excess in China shows no clear pattern with extreme high values (>14‰) found in northwestern arid region (including Xinjiang, Gansu, Qinghai provinces). This is the same finding for extreme low values (<1‰) found in North China Plain and Inner Mongolia, except for one specific city named Xichang (−0.77‰) located on Yunnan-Guizhou Plateau in southwestern China (see the dark brown color site in Fig. 1).

Standard deviation of monthly isotope values of each site were calculated in order to analyze intra-annual variability of tap water in China. The standard deviation values range from 0.06‰ to 1.79‰, 0.12‰ to 11.83‰ and 0.01‰ to 6.46‰ for δ^18^O, δ^2^H and d-excess, respectively (Table 2). In general, intra-annual variability shows no clear spatial pattern. For certain areas, isotope values exhibit low intra-annual variability, such as Inner Mongolia, Gansu and Qinghai provinces. Sample locations with relatively high intra-annual variability mainly occurred in coastal regions. Similar to δ^2^H and δ^18^O, intra-annual variability of deuterium excess exhibits no clear spatial pattern. Extreme standard deviation value (6.46‰) occurred in Xichang. Moreover, sampling locations with relatively high intra-annual variability centered in western part of the country ranging from 2.5‰ to 3.5‰.

### Temporal variability of tap water isotopes

Temporal variability of isotopes in individual tap water sampling locations was evaluated based on monthly dataset. However, due to certain unavoidable factors including human factors and express delivery’s delay in sending and receiving sampling bottles, interval of tap sample acquisition wasn’t exactly 30 days but varied from 20 to 40 days. Sampling data series weren’t sequential at monthly scale. Therefore, temporal variability was calculated by on-site seasonal comparison: spring (average of March, April and May in 2015) minus winter (average of December in 2014, January and February in 2015), summer (average of June, July and August in 2015) minus spring, autumn (average of September, October and November in 2015) minus summer (see data statistics in Table 3).

**Figure 4:**
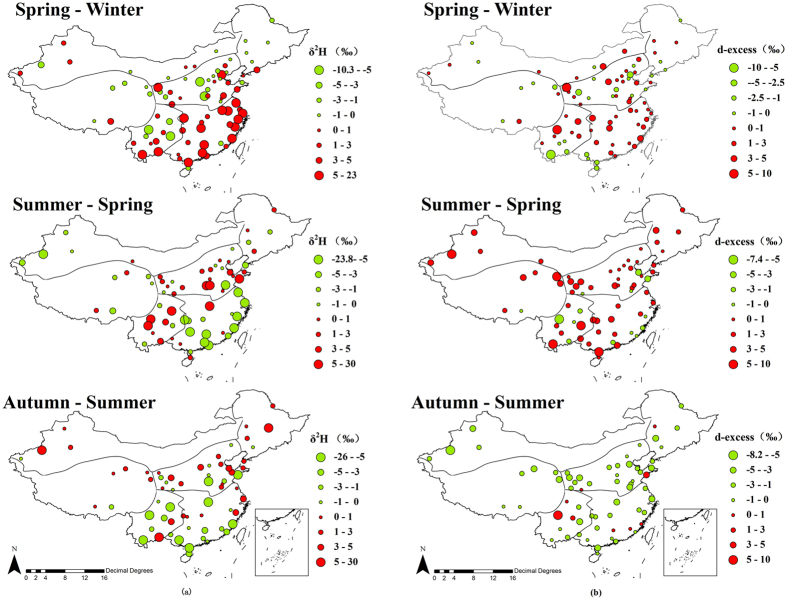
Seasonal differences of δ^2^H and d-excess values in tap water, expressed in circles with different sizes and two colors, green represents value decreasing and red represents increasing, circle size represents the magnitude of seasonal variation. (All of the items were generated with Arcgis 9.3, https://www.arcgis.com/features/index.html).

Seasonal differences of δ^2^H isotope values spanned 48.51‰ (−25.99‰ to 22.52‰) with an average of 0.38‰ and a standard deviation of 5.29‰. Seasonal differences of δ^18^O isotope value spanned 5.88‰ (−2.99‰ to 2.89‰), with an average of 0.02‰ and a standard deviation of 0.78‰. At national scale, there seems no specific pattern of seasonal variability. However, detailed interpretations of seasonal patterns can be found at the regional scale, which is consistent with the findings in precipitation isotope across China by Chen *et al*.^37^ (Fig. 4(a)). Considering the relationship between δ^2^H and δ^18^O, only the δ^2^H plots are shown.

In southeastern regions (Guangxi, Guangdong, Jiangsu, Zhejiang, Shanghai, Fujian, Anhui, Jiangxi, Hunan, Hubei) with a total number of 27 samples locations, most sample locations experienced isotope values rose from winter to spring and dropped from spring to autumn. In general, the maximum isotope values of southeastern region usually occurred in spring and the minimum values occurred in summer or winter.

In northeastern China (Heilongjiang province, Jilin province, Liaoning province and northeast of Inner Mongolia) with 8 samples locations, isotope values in all samples locations except Dalian and Dandong (see rose quartz and apple green color site in Fig. 1) reached the lowest point in late spring or early summer (May or June) and increased to top in late autumn or early winter (November or December) with a spanning range of 6.95‰ in average.

Different from the first-sight-guess that extreme isotope values should occur in summer or winter with difference value spanning a large range, for example, stable isotopes in precipitation present regular temporal trends driven by monsoon^37^. Seasonal variability of isotopes in tap water exhibits various pattern with extreme values occurring in various seasons. The reasons might be: a) tap water has mixing water sources as compared to precipitation; and b) there is a lag time between tap water and precipitation. Although only 6 locations on Tibet Plateau provided tap water samples, seasonal trend of isotopes in 5 locations except Nyingchi (see ginger pink color site in Fig. 1) exhibited similar pattern with isotope values decreasing from winter to spring and increasing from summer to autumn. Many factors could contribute to this trend including geographical, climatic, and hydrological factors. Compared to warm regions, the hydrological factors influencing SITW in Tibet Plateau are more complex due to its unique and comprehensive processes happening in cold area, e.g., snow and glacier melting^38^,^39^,^40^,^41^.

These results mean intra-annual variabilities of isotope ratios in tap water are relatively large and the temporal patterns of different regions divided according to the spatial pattern are significantly different. In other words, the temporal patterns of isotopic compositions are, to some extent, correlated with spatial pattern.

Seasonal differences of deuterium excess value spanned 15.61‰ (−8.12‰ to 7.49‰) with an average of 2.44‰ and a standard deviation of 2.36‰. Deuterium excess is known as providing information about climate conditions of water moisture^42^. Seasonal variability of d-excess is presented in Fig. 4(b). On national scale, deuterium excess values in 76% of the locations increased all the way from winter to summer for about 2.03‰ in average and dropped from summer to autumn for about 1.69‰ in average. Special sample locations with different variation patterns included Heihe in northeast, Korla and Karamay in northwest, 11 locations in north China, Lhasa and Nyingchi on Tibet Plateau and 9 locations in southwest (see color site in Fig. 1). Tap water grabbed from winter or autumn exhibited the most extreme negative d-excess values and lay furthest from GMWL, suggesting a strong evaporative isotopic fractionation of the source waters. While tap samples from summer obtaining the highest d-excess values suggested more evaporated moisture has been added to the atmosphere^43^.

### Correlations between isotope values in tap water and environmental variables

Given that isotopes in tap water present various spatial and temporal patterns across China, more detailed work was conducted to analyze environmental factors influencing tap water isotopes. As demonstrated in many previous studies, isotopes in precipitation^23^,^44^,^46^ or river^16^,^46^ are strongly correlated to geographical factors (e.g. longitude, latitude, elevation) and climatic factors (e.g., air temperature, precipitation, relative humidity and air pressure *et al*.) However, tap water does not directly get involved in natural water circulation processes like precipitation, surface water or groundwater. It is a mixture of locally available waters (including rivers, lakes, wells and springs). Therefore, interpretation of tap water isotopes and environmental variables may not be similar to precipitation, which presents ‘temperature effect’ resulting from different processes of isotopic fractionation^29^.

**Figure 5:**
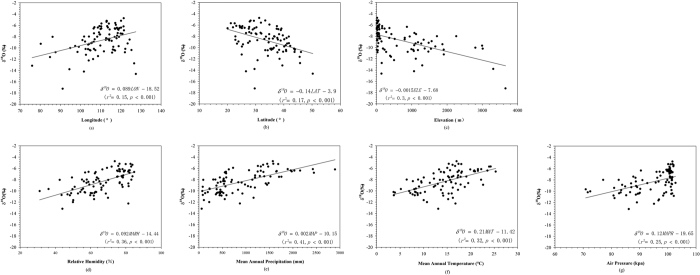
Correlations between mean annual observed δ^18^O and (**a**) longitude (°), (**b**) latitude (°), (**c**) elevation (m), (**d**) mean annual relative humidity (MARH, %), (**e**) mean annual precipitation (MAP, mm), (**f**) mean annual temperature (MAT, °C), (**g**) mean annual air pressure (MAPR, kpa). All isotopes data and meteorological data are means between December 2014 and December 2015.

Figure 5 illustrates correlations between mean annual values of δ^18^O and mean annual values of climatic variables or geographic parameters. Note that the elevation data used here is taken from station observation provided by China Meteorological Data System (Table 1). Even though spatial pattern (“continental effect”) of isotopes can appear in tap water, the coefficient of determination between δ^18^O and longitude, latitude and elevation were low (r^2^ = 0.15, 0.17 and 0.3 for longitude, latitude and elevation, respectively, p < 0.001 for all cases) (Fig. 5(a–c)). Nonetheless, the slope of regression line that reflects elevation effect is −0.15‰/100 m, which compared well with results of China precipitation δ^18^O values demonstrated by Liu *et al*.^23^ (−0.13‰/100 m for height).

Correlations between isotopic composition and meteorological factors have been analyzed with 4 extreme low locations (Lhasa, Nyingchi, Heihe and Harbin mentioned in section 3.1) left out (Fig. 5(d–g)). tap water δ^18^O across China had a relatively strong positive correlation with mean annual precipitation (MAP), mean annual temperature (MAT) and mean annual relative humidity (MARH) (r^2^ = 0.41, 0.32 and 0.36 for MAP, MAT and MARH, respectively, p < 0.001 for all cases). The correlation with mean annual air pressure (MAPR) was weaker (r^2^ = 0.25, p < 0.001). The δ^18^O-temperature gradient was 0.21‰/°C, lower than values of China precipitation with a range between 0.27‰/°C and 0.58‰/°C^23^.

Based on these relations, a multiple regression model on national scale can be obtained as


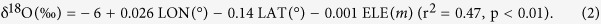


Considering climatic parameters, multiple regression model can be expressed as


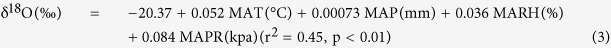


Similarly, multiple regression model for tap water δ^2^H is as follows:


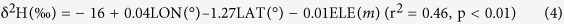



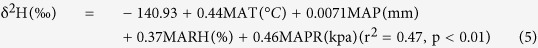


Correlations between mean annual d-excess and environmental variables were also analyzed. However, there are no significant correlations between d-excess and those seven environmental factors with all correlation coefficients lower than 0.1. D-excess in air masses (and hence precipitation) depends on the relative humidity of the air masses at their oceanic origin, the ocean surface temperature, and kinetic isotope effects during evaporation^47^. Given this, it is expected that correlations between d-excess and other environmental factors are weak. In addition, “mixing effect”, involving different natural water sources, can also smear the signature leading to such results.

The limitations of this work arise from the data constraints, and the complexity of the natural water cycle and tap water system. First, tap water isotope data requires improvements in sampling duration and spatial coverage to better represent the spatial and temporal pattern across the whole country. This is especially true for the seasonal variability analysis and multi-year observations are preferable. Therefore, current analyses on temporal variability at the seasonal scale might need further refinement. Second, because of the difficulty in sampling concurrent precipitation, surface water, groundwater and examining the complex tap water processing system, we can hardly trace the initial origin of tap water and thus decouple all the mixing signature based on the current data. In this regard, correlations with environmental factors may be informative, but not ideal to investigate the controlling factors of tap water stable isotopic compositions. Further work is needed to better understand the impact of human activity on drinking water system.

## Conclusion

To our best knowledge, this study is the first to report tap water isotopic composition over China, which was achieved by establishing a nation-wide volunteer network. Result demonstrated that SITW spatial pattern presents “continental effect” with a decreasing trend in isotopic compositions from coastal regions with low latitude and elevation to inland regions with high latitude and elevation. SITW seasonal trend indicates clearly regional patterns but no trends at the national level, which is consistent with spatial pattern. Also, there are positive correlations between mean annual isotope values and meteorological parameters including precipitation, temperature, relative humidity and air pressure. Correlations between isotope values and geographic factors taken individually are relatively weak but through multiple regression model, the combined geographic factors explain a large variability in isotopic compositions. This work presents the first SITW map in China and establishes a benchmark for further stable isotope research across China.

## Additional Information

**How to cite this article:** Zhao, S. *et al*. Divergence of stable isotopes in tap water across China. *Sci. Rep.* **7**, 43653; doi: 10.1038/srep43653 (2017).

**Publisher's note:** Springer Nature remains neutral with regard to jurisdictional claims in published maps and institutional affiliations.

## Figures and Tables

**Table 1:** Listing of geographical and meteorological information of each sampling locations, including latitude, longitude and elevation above sea level in meters, mean annual temperature (MAT), precipitation (MAP), relative humidity (MARH) and air pressure (MAPR).

No	Sample Location	Latitude	Longtitude	Elvation	MAP (mm)	MAT (°C)	MARH (%)	MAPR (Kpa)
**1**	Heihe	50.24	127.49	139	679.2	2.39	66.12	98.56
**2**	Harbin	45.74	126.64	147	414	5.61	64.19	100.03
**3**	Karamay	45.60	84.86	410	106.4	10.01	50.13	96.68
**4**	Urumchi	43.79	87.61	883	585.5	3.67	59.79	80.75
**5**	Aksu	41.17	80.26	1107	82.2	12.17	50.96	89.13
**6**	Korla	41.76	86.13	940	92.7	13.46	42.47	91.01
**7**	Kashgar	39.47	75.99	1296	59.6	13.11	43.98	86.16
**8**	Jiuquan	39.74	98.51	1470	83.4	8.75	43.53	85.25
**9**	Delingha	37.37	97.36	2995	229.3	5.16	36.14	70.89
**10**	Golmud	36.43	94.89	2803	72.6	6.92	31.10	72.50
**11**	Xining	36.61	101.79	2261	304.9	6.40	55.11	77.09
**12**	Lanzhou	36.07	103.75	1543	189.2	8.32	56.60	83.30
**13**	Baiyin	36.54	104.18	1713	350.2	8.82	57.79	82.59
**14**	Baotou	40.67	109.85	1068	225.7	8.35	55.75	90.23
**15**	Hohhot	40.82	111.66	1057	361.1	7.66	46.90	88.66
**16**	Linhe	40.76	107.39	1040	133.5	8.71	48.95	89.81
**17**	Yinchuan	38.47	106.27	1113	219.8	10.71	49.96	89.06
**18**	Yulin	38.30	109.76	1121	445.7	10.03	49.42	88.56
**19**	Taiyuan	37.87	112.57	797	401.2	11.32	57.64	92.74
**20**	Jinzhong	37.68	112.75	800	515.8	7.71	64.97	88.67
**21**	Shijiazhuang	38.05	114.49	80	552.2	14.36	56.87	101.06
**22**	Anyang	36.10	114.35	78	463.2	14.72	58.73	99.38
**23**	Pingliang	35.54	106.68	1365	487	10.28	62.72	86.64
**24**	Ulanhot	46.07	122.07	273	440.2	8.12	48.24	98.37
**25**	Xilinhot	43.94	116.07	988	408.4	3.89	56.90	90.09
**26**	Tongliao	43.61	122.26	181	475.6	8.17	54.18	99.42
**27**	Changchun	43.89	125.32	227	520.8	7.17	60.09	98.71
**28**	Chifeng	42.27	118.95	572	377.6	8.00	49.68	93.91
**29**	Shenyang	41.80	123.41	50	564.8	9.02	61.66	101.06
**30**	Chengde	40.97	117.92	361	549.6	9.49	56.16	96.72
**31**	Dandong	40.14	124.38	18	908.9	9.55	68.74	102.73
**32**	Beijing	40.12	116.30	31	456.8	13.69	54.89	101.32
**33**	Tianjin	39.13	117.20	9	576	13.66	58.82	101.69
**34**	Tangshan	39.63	118.20	23	527.9	12.20	64.37	101.43
**35**	Baoding	38.86	115.50	21	534.4	13.00	65.66	101.51
**36**	Cangzhou	38.31	116.86	8	715	14.05	60.80	101.58
**37**	Dalian	38.92	121.60	21	579.6	12.11	62.42	100.60
**38**	Hengshui	37.73	115.71	26	480.4	14.17	61.79	102.12
**39**	Dongying	37.46	118.50	5	560.6	14.30	62.89	101.65
**40**	Yantai	37.54	121.38	43	636.3	13.28	66.67	100.74
**41**	Weifang	36.70	119.11	28	523.1	14.10	63.13	101.48
**42**	Lhasa	29.66	91.13	3657	339.7	9.47	34.38	65.32
**43**	Gannan	35.20	102.51	3012	447.3	3.60	63.18	71.61
**44**	Dingxi	35.58	104.62	1905	382.6	9.33	62.15	82.72
**45**	Longnan	33.39	104.93	1174	450	15.97	52.84	89.36
**46**	Chengdu	30.66	104.08	497	872.3	16.78	81.39	95.11
**47**	Nyingchi	29.58	94.48	3310	934	9.44	62.83	71.02
**48**	Xichang	27.90	102.27	1563	980.8	17.87	59.27	83.81
**49**	Panzhihua	26.55	101.70	1064	1049.9	21.20	56.77	87.46
**50**	Baoshan	25.12	99.17	1667	832.4	17.31	66.11	83.47
**51**	Kunming	25.04	102.70	1907	1162.9	16.22	69.87	81.11
**52**	Qujing	25.50	103.79	1868	1191.1	16.20	66.18	80.98
**53**	Simao	22.80	100.98	1336	1482	19.49	76.45	86.98
**54**	Wenshan	23.37	104.24	1268	1103.1	18.75	78.15	87.07
**55**	Tianshui	34.58	105.72	1176	372.6	12.47	67.18	88.76
**56**	Zhengzhou	34.76	113.65	106	688.2	15.87	61.64	100.40
**57**	Kaifeng	34.79	114.35	70	581.9	15.42	63.56	100.83
**58**	Hanzhong	33.08	107.03	515	838.7	15.88	76.54	95.71
**59**	Xianyang	34.34	108.71	384	550	15.22	61.97	96.88
**60**	Enshi	30.27	109.48	421	1193.3	17.23	78.32	96.27
**61**	Wuhan	30.57	114.29	16	1421.1	16.83	81.41	101.33
**62**	Chongqing	29.56	106.51	157	1250.3	18.12	78.89	96.00
**63**	Yueyang	29.37	113.10	46	1687.1	18.02	79.90	100.97
**64**	Changsha	28.20	112.98	54	1452.8	17.42	82.63	100.14
**65**	Bijie	27.31	105.28	1478	1043.5	14.04	81.55	84.88
**66**	Zunyi	27.70	106.93	861	1066.1	15.63	82.18	90.46
**67**	Tongren	27.72	109.19	274	1195	17.35	78.51	97.41
**68**	Huaihua	27.55	109.95	227	1372	17.21	84.72	98.36
**69**	Hong Kong	27.87	112.92	43	1365.7	16.16	83.72	102.05
**70**	Guiyang	26.58	106.71	1073	1390	15.20	83.75	87.78
**71**	Guilin	25.28	110.29	160	2894.7	19.92	76.53	99.49
**72**	Zaozhuang	34.87	117.56	80	727.1	15.03	69.30	100.80
**73**	Xuzhou	34.27	117.19	35	925	15.31	68.63	101.21
**74**	Suzhou	33.64	116.97	37	702.1	15.64	69.97	101.39
**75**	Yancheng	33.39	120.14	5	1582.8	15.34	76.38	101.68
**76**	Nantong	32.02	120.86	11	1705.1	16.01	77.92	101.64
**77**	Hefei	31.86	117.28	22	1254.4	16.70	75.68	101.98
**78**	Ma’anshan	31.72	118.48	29	1295.8	16.33	77.64	100.73
**79**	Shanghai	31.24	121.47	16	1573.2	17.01	73.71	101.64
**80**	Shaoxing	30.01	120.57	11	1755.7	17.86	75.03	101.56
**81**	Hangzhou	30.27	120.16	18	2030.6	17.49	75.04	101.16
**82**	Quzhou	28.96	118.87	79	2446.2	18.02	82.09	100.62
**83**	Lishui	28.45	119.92	64	1522.8	19.09	75.34	100.88
**84**	Fuzhou	26.08	119.30	18	1655.4	20.69	75.48	100.54
**85**	Longyan	25.11	117.03	365	1975.4	20.92	76.59	97.11
**86**	Liuzhou	24.31	109.40	65	1889.1	21.55	76.23	100.19
**87**	Shaoguan	24.81	113.61	65	1953.8	20.78	81.64	100.02
**88**	Xiamen	24.46	118.09	31	1316.1	21.55	79.01	99.81
**89**	Bose	23.90	106.61	141	1450.2	22.65	77.79	99.15
**90**	Guangzhou	23.12	113.26	28	2424	22.26	78.09	100.52
**91**	Nanning	22.81	108.31	80	1136.2	22.23	83.12	99.83
**92**	Shenzhen	22.56	114.11	8	1473.6	23.93	71.91	100.60
**93**	Qinzhou	21.95	108.61	10	2153	23.57	79.45	100.84
**94**	Zhanjiang	21.19	110.40	17	1316.2	24.26	83.00	100.59
**95**	Haikou	20.03	110.35	15	1646	25.33	81.35	100.44

**Table 2:** Summary of δ^18^O and δ^2^H values in tap water samples and d-excess =  ^2^H–8δ^18^O.

No	Sample site	count	δ^18^O (‰)				δ^2^H (‰)				d-excess (‰)			
			Max	Min	std	Average	Max	Min	std	Average	Max	Min	std	Average
1	Heihe	9	−13.88	−15.93	0.58	−14.64	−103.11	−119.31	4.93	−108.68	11.90	7.00	1.53	8.48
2	Harbin	13	−12.20	−13.46	0.43	−12.72	−88.71	−96.62	2.54	−92.56	11.89	7.75	1.32	9.18
3	Karamay	10	−11.23	−12.38	0.33	−11.63	−75.23	−79.42	1.20	−77.20	19.60	14.64	1.80	15.85
4	Urumchi	3	−10.66	−10.86	0.08	−10.77	−72.33	−73.48	0.48	−72.83	13.70	12.91	0.32	13.32
5	Aksu	8	−7.73	−11.25	1.17	−9.28	−56.86	−72.46	5.23	−63.41	17.56	4.94	4.34	10.80
6	Korla	12	−7.44	−10.48	0.77	−8.04	−54.63	−70.61	4.13	−57.31	13.20	4.90	2.11	7.02
7	Kashgar	10	−12.62	−13.96	0.45	−13.17	−88.37	−94.82	2.04	−91.19	18.41	11.78	2.42	14.20
8	Jiuquan	6	−10.53	−10.92	0.13	−10.73	−73.87	−75.12	0.42	−74.53	12.47	10.34	0.68	11.28
9	Delingha	10	−9.16	−10.11	0.29	−9.67	−58.89	−64.84	1.84	−61.76	19.11	14.09	1.77	15.60
10	Golmud	10	−9.60	−10.56	0.36	−10.03	−64.78	−67.99	0.91	−66.60	16.96	11.08	2.09	13.65
11	Xining	9	−6.75	−8.70	0.55	−7.99	−44.16	−48.97	1.34	−47.21	20.60	9.87	3.21	16.74
12	Lanzhou	6	−10.02	−10.54	0.16	−10.22	−69.97	−75.37	1.70	−72.01	11.37	8.80	0.86	9.77
13	Baiyin	12	−6.49	−10.49	1.19	−7.99	−42.03	−73.22	10.89	−51.13	16.21	5.10	3.07	12.83
14	Baotou	5	−9.12	−9.95	0.34	−9.59	−66.50	−72.78	2.77	−70.12	7.32	5.98	0.49	6.57
15	Hohhot	5	−10.08	−10.93	0.31	−10.62	−77.41	−80.60	1.20	−78.20	9.60	0.04	3.51	6.73
16	Linhe	2	−9.87	−10.94	0.53	−10.41	−75.05	−79.33	2.14	−77.19	8.16	3.95	2.10	6.06
17	Yinchuan	5	−11.86	−12.08	0.08	−12.00	−84.27	−85.52	0.46	−85.08	11.11	10.63	0.19	10.89
18	Yulin	6	−7.43	−7.78	0.14	−7.62	−59.42	−61.44	0.78	−60.57	0.82	−0.27	0.39	0.41
19	Taiyuan	5	−8.24	−9.20	0.35	−8.57	−62.41	−68.00	2.09	−63.98	5.61	3.48	0.84	4.57
20	Jinzhong	10	−7.29	−8.87	0.57	−8.28	−56.54	−63.82	2.36	−60.48	9.75	1.79	2.60	5.74
21	Shijiazhuang	7	−6.42	−7.02	0.20	−6.74	−49.79	−54.11	1.33	−51.90	3.54	0.69	0.90	2.00
22	Anyang	8	−8.21	−8.47	0.08	−8.40	−53.51	−61.29	2.36	−60.16	12.16	5.78	1.85	7.04
23	Pingliang	10	−9.42	−10.71	0.46	−10.18	−65.56	−72.53	2.27	−69.06	16.45	9.06	2.43	12.36
24	Ulanhot	12	−10.31	−11.28	0.32	−10.78	−79.50	−84.39	1.06	−81.32	8.78	2.42	2.26	4.94
25	Xilinhot	5	−10.07	−10.50	0.15	−10.29	−80.73	−82.83	0.73	−82.10	1.42	−0.54	0.76	0.24
26	Tongliao	8	−9.87	−10.75	0.27	−10.18	−77.97	−80.18	0.76	−78.93	6.22	0.85	1.55	2.49
27	Changchun	3	−10.24	−10.55	0.13	−10.38	−79.90	−80.20	0.12	−80.06	4.18	1.84	0.96	2.96
28	Chifeng	10	−8.59	−10.06	0.41	−9.70	−70.16	−77.51	1.83	−74.70	5.71	−1.45	1.77	2.89
29	Shenyang	13	−8.94	−9.58	0.20	−9.22	−65.82	−69.36	1.17	−67.33	7.41	5.39	0.65	6.39
30	Chengde	4	−8.23	−8.43	0.08	−8.30	−62.04	−63.17	0.45	−62.40	4.30	3.65	0.25	3.96
31	Dandong	6	−7.73	−8.89	0.40	−8.53	−53.30	−61.28	2.71	−58.86	10.37	8.45	0.70	9.35
32	Beijing	12	−9.38	−10.37	0.30	−9.81	−62.95	−67.89	1.68	−65.59	15.04	11.44	0.89	12.87
33	Tianjin	4	−5.76	−7.03	0.50	−6.62	−49.04	−55.25	2.44	−53.15	0.96	−2.94	1.60	−0.19
34	Tangshan	4	−7.69	−7.95	0.10	−7.83	−57.13	−58.12	0.40	−57.67	5.70	4.15	0.69	4.96
35	Baoding	7	−8.38	−9.06	0.24	−8.75	−62.37	−65.27	1.06	−63.81	7.23	4.69	0.91	6.22
36	Cangzhou	13	−9.38	−10.75	0.33	−10.40	−75.08	−78.65	0.89	−77.41	7.69	−0.07	1.82	5.81
37	Dalian	10	−6.37	−7.47	0.36	−6.89	−49.28	−54.98	1.96	−52.02	5.51	1.72	1.07	3.10
38	Hengshui	6	−10.59	−10.99	0.12	−10.84	−79.35	−81.31	0.64	−79.92	7.57	5.39	0.73	6.82
39	Dongying	8	−5.33	−7.39	0.75	−6.66	−45.28	−56.23	3.75	−51.95	3.84	−2.62	2.39	1.31
40	Yantai	9	−5.50	−6.81	0.38	−6.19	−44.09	−49.00	1.71	−46.69	6.62	−0.12	1.83	2.84
41	Weifang	6	−7.49	−8.46	0.34	−8.04	−56.04	−61.37	1.83	−59.10	6.49	3.92	0.98	5.23
42	Lhasa	12	−16.49	−17.74	0.38	−17.26	−122.99	−132.09	2.75	−129.47	13.01	5.39	1.80	8.58
43	Gannan	10	−9.90	−10.80	0.28	−10.20	−66.80	−69.49	0.80	−68.26	17.40	11.58	1.85	13.31
44	Dingxi	10	−10.04	−10.98	0.28	−10.38	−67.64	−71.77	1.37	−69.97	17.50	10.80	2.27	13.04
45	Longnan	7	−10.22	−10.68	0.14	−10.45	−68.60	−69.99	0.47	−69.23	15.46	13.18	0.68	14.41
46	Chengdu	11	−11.95	−13.17	0.33	−12.25	−81.70	−87.21	1.47	−83.83	18.12	12.19	1.55	14.15
47	Nyingchi	10	−12.58	−14.38	0.56	−13.80	−87.42	−104.03	5.29	−98.49	13.78	10.87	0.91	11.91
48	Xichang	11	−7.34	−12.97	1.79	−8.87	−63.42	−90.67	8.03	−71.77	13.12	−5.86	6.46	−0.77
49	Panzhihua	5	−13.49	−14.63	0.39	−14.21	−96.87	−108.98	4.30	−105.09	11.02	7.32	1.34	8.60
50	Baoshan	6	−9.63	−10.22	0.19	−9.81	−68.47	−72.78	1.56	−69.30	9.78	8.57	0.44	9.18
51	Kunming	9	−10.92	−11.92	0.30	−11.23	−81.09	−85.70	1.27	−82.66	10.04	5.78	1.48	7.16
52	Qujing	3	−9.06	−9.84	0.32	−9.42	−68.62	−73.58	2.03	−71.20	5.13	3.56	0.68	4.17
53	Simao	10	−7.38	−10.20	0.91	−8.62	−55.32	−69.16	5.14	−62.29	12.44	3.02	2.90	6.63
54	Wenshan	8	−6.39	−10.80	1.31	−9.30	−49.35	−76.26	7.98	−66.50	10.16	1.80	2.56	7.87
55	Tianshui	10	−8.61	−9.64	0.32	−9.08	−58.07	−61.56	1.00	−60.06	15.77	10.16	1.96	12.54
56	Zhengzhou	7	−7.82	−8.89	0.40	−8.42	−54.73	−64.17	3.99	−59.74	10.48	5.96	1.36	7.60
57	Kaifeng	7	−5.98	−8.32	0.80	−7.61	−36.40	−60.71	8.06	−55.36	11.46	0.95	2.90	5.56
58	Hanzhong	12	−7.48	−9.04	0.41	−8.48	−47.63	−59.40	3.21	−54.99	13.85	11.97	0.59	12.83
59	Xianyang	11	−11.08	−11.80	0.22	−11.47	−81.35	−86.57	1.54	−83.57	9.91	5.80	1.16	8.18
60	Enshi	5	−6.30	−8.28	0.72	−7.62	−34.56	−60.80	8.70	−48.44	15.88	5.48	3.69	12.53
61	Wuhan	9	−7.62	−9.44	0.56	−8.69	−47.68	−65.66	5.41	−58.10	13.30	9.67	1.16	11.38
62	Chongqing	11	−4.34	−9.22	1.48	−6.57	−22.98	−60.67	11.83	−41.47	15.78	7.10	2.32	11.08
63	Yueyang	5	−4.59	−4.70	0.04	−5.25	−26.60	−30.55	1.46	−28.86	10.13	6.37	1.33	8.50
64	Changsha	5	−4.76	−5.52	0.26	−5.21	−25.56	−33.72	2.88	−30.78	14.18	8.98	2.11	10.87
65	Bijie	11	−8.43	−9.72	0.38	−9.16	−55.45	−64.65	3.06	−60.88	15.13	9.87	1.21	12.41
66	Zunyi	1	−6.34	−6.34	0.00	−6.34	−41.09	−41.09	0.00	−41.09	9.59	9.59	0.00	9.59
67	Tongren	10	−6.52	−8.29	0.62	−7.25	−38.10	−49.04	3.37	−43.60	17.55	11.44	2.02	14.41
68	Huaihua	9	−5.81	−7.56	0.57	−6.65	−33.91	−45.41	3.33	−40.74	17.03	3.74	3.67	12.42
69	Hong Kong	10	−4.85	−5.99	0.39	−5.38	−28.10	−33.25	1.38	−31.71	15.20	8.94	2.28	11.31
70	Guiyang	8	−6.00	−8.50	0.93	−7.42	−41.96	−55.02	4.88	−49.69	14.58	6.04	3.42	9.65
71	Guilin	13	−5.63	−7.25	0.56	−6.19	−32.39	−42.30	3.40	−36.37	16.88	10.88	1.69	13.18
72	Zaozhuang	13	−4.76	−7.69	0.81	−5.91	−39.45	−51.91	4.03	−45.88	9.64	−1.52	2.65	2.85
73	Xuzhou	12	−10.16	−11.16	0.24	−10.48	−75.35	−77.36	0.54	−76.46	11.88	5.97	1.51	7.37
74	Suzhou	9	−6.98	−8.37	0.40	−8.05	−54.38	−58.28	1.12	−57.36	8.68	1.43	2.09	7.07
75	Yancheng	13	−4.05	−6.77	0.70	−5.19	−28.06	−45.65	4.69	−37.20	8.53	1.29	1.88	4.29
76	Nantong	5	−6.52	−8.17	0.77	−7.51	−40.77	−56.79	7.29	−50.29	12.28	8.01	1.45	9.82
77	Hefei	5	−5.13	−5.93	0.36	−5.58	−32.99	−41.08	3.55	−37.67	8.02	5.61	0.99	6.98
78	Ma’anshan	5	−6.50	−7.92	0.52	−7.37	−42.35	−53.14	4.06	−49.15	10.81	8.36	0.81	9.78
79	Shanghai	10	−3.80	−6.17	0.68	−4.74	−24.10	−37.92	3.97	−30.69	11.47	4.87	2.00	7.19
80	Shaoxing	6	−6.93	−7.98	0.33	−7.49	−42.07	−53.21	3.91	−48.58	13.37	9.34	1.51	11.36
81	Hangzhou	2	−6.35	−6.46	0.06	−6.40	−39.75	−40.61	0.43	−40.18	11.05	11.02	0.01	11.04
82	Quzhou	13	−6.28	−7.26	0.26	−6.79	−38.09	−46.50	2.50	−42.46	14.41	10.10	1.45	11.85
83	Lishui	9	−6.67	−8.36	0.51	−7.45	−39.29	−54.74	4.57	−47.29	14.09	9.78	1.26	12.35
84	Fuzhou	11	−5.74	−7.54	0.62	−6.49	−32.00	−48.53	5.48	−40.50	14.33	6.37	2.03	11.40
85	Longyan	7	−6.31	−6.75	0.12	−6.54	−37.16	−40.36	1.05	−38.85	14.30	12.68	0.55	13.49
86	Liuzhou	2	−5.96	−6.32	0.18	−6.14	−33.59	−36.75	1.58	−35.17	14.06	13.79	0.14	13.93
87	Shaoguan	13	−5.39	−7.61	0.69	−6.38	−30.01	−44.33	3.96	−38.23	16.53	9.45	2.23	12.81
88	Xiamen	10	−5.85	−7.44	0.49	−6.43	−36.71	−46.74	3.60	−40.38	13.62	9.90	1.15	11.06
89	Bose	9	−8.39	−9.86	0.50	−8.92	−57.36	−65.20	2.07	−60.41	14.67	8.22	2.44	10.93
90	Guangzhou	9	−4.86	−6.90	0.60	−6.14	−26.02	−45.69	5.66	−38.86	12.83	9.27	1.14	10.29
91	Nanning	11	−7.44	−8.66	0.36	−8.02	−49.04	−60.50	3.05	−55.35	10.49	7.46	0.85	8.79
92	Shenzhen	9	−4.80	−7.15	0.74	−5.69	−28.86	−43.65	4.48	−35.60	13.52	8.11	1.81	9.92
93	Qinzhou	5	−5.97	−6.93	0.38	−6.40	−36.32	−44.15	2.99	−40.18	11.83	10.22	0.54	10.99
94	Zhanjiang	8	−4.01	−8.08	1.36	−5.68	−26.91	−53.91	9.86	−38.54	10.74	4.60	2.29	6.87
95	Haikou	7	−6.31	−6.78	0.15	−6.57	−41.84	−45.47	1.32	−44.17	9.46	6.95	0.77	8.38

**Table 3:** Summary of δ^18^O, δ^2^H and d-excess seasonal values in tap water samples. NE, NW,N,SW,SE,QP stands for stands for different regions of China namely northeastern, northwestern, northern, southwestern, southeastern and Qinghai-Tibet Plateau.

Sample Location	Region	δ^18^O (‰)				δ^2^H (‰)				d-excess (‰)			
		winter	spring	summer	autumn	winter	spring	summer	autumn	winter	spring	summer	autumn
Heihe	NE	−14.73	−14.77	−14.78	−14.40	−109.69	−110.73	−108.27	−106.58	8.18	7.40	9.97	8.65
Harbin	NE	−12.59	−12.77	−13.29	−12.27	−92.47	−93.10	−95.07	−89.62	8.23	9.05	11.21	8.55
Karamay	NW	−11.56	−11.39	−12.01	−11.52	−77.52	−76.17	−78.00	−77.34	14.99	14.95	18.07	14.83
Urumchi	NW	−10.77				−72.83				13.32			
Aksu	NW	−8.20	−8.70	−10.57	−8.21	−58.09	−61.14	−69.12	−58.40	7.52	8.43	15.42	7.31
Korla	NW	−8.33	−7.84	−8.07	−7.71	−59.23	−56.30	−57.01	−55.45	7.44	6.45	7.55	6.25
Kashgar	NW	−13.06	−12.72	−13.58	−13.34	−91.75	−89.02	−92.01	−92.36	12.71	12.72	16.61	13.77
Jiuquan	NW	−10.63	−10.77	−10.92		−74.16	−74.93	−74.86		10.89	11.27	12.47	
Delingha	QP	−9.54	−9.84	−9.90	−9.32	−61.44	−63.94	−61.52	−59.94	14.85	14.76	17.66	14.62
Golmud	QP	−9.63	−9.80	−10.40	−10.02	−64.78	−66.24	−67.34	−66.82	12.29	12.19	15.88	13.34
Xining	QP	−7.70	−7.53	−8.44	−8.17	−45.97	−46.33	−48.32	−47.48	15.62	13.90	19.20	17.85
Lanzhou	N	−10.21	−10.29		−10.13	−72.08	−72.11		−71.82	9.57	10.18		9.26
Baiyin	N	−9.73	−7.64	−7.50	−6.98	−69.07	−46.55	−44.81	−44.09	8.81	14.58	15.20	12.72
Baotou	NW	−9.62	−9.54			−70.28	−69.88			6.68	6.40		
Hohhot	NW	−10.42	−10.91			−78.52	−77.72			4.82	9.59		
Linhe	NW	−10.41				−77.19				6.06			
Yinchuan	NW	−11.95	−12.03			−84.73	−85.32			10.87	10.91		
Yulin	N	−7.61	−7.64			−60.41	−60.73			0.44	0.38		
Taiyuan	N	−8.47	−8.72			−63.17	−65.20			4.59	4.55		
Jinzhong	N	−7.53	−8.68	−8.61	−7.79	−57.77	−62.91	−61.54	−57.89	2.50	6.57	7.33	4.39
Shijiazhuang	N	−6.80	−6.93	−6.71	−6.42	−52.84	−52.93	−50.62	−50.64	1.57	2.47	3.07	0.69
Anyang	N	−8.34	−8.42	−8.45	−8.39	−58.68	−61.05	−60.65	−60.91	8.06	6.35	6.94	6.23
Pingliang	N	−10.71	−10.01	−10.58	−9.77	−72.53	−69.63	−69.86	−66.53	13.11	10.42	14.75	11.66
Ulanhot	NE	−10.55	−10.68	−10.98	−10.92	−81.12	−82.05	−81.41	−80.72	3.27	3.39	6.43	6.66
Xilinhot	NW	−10.19	−10.44			−81.91	−82.37			−0.36	1.13		
Tongliao	NE	−9.93	−10.07	−10.47	−9.87	−77.97	−78.59	−79.84	−78.14	1.50	1.96	3.90	0.85
Changchun	NE	−10.38				−80.06				2.96			
Chifeng	N	−9.45	−9.73	−9.89	−9.80	−74.10	−74.70	−75.09	−75.34	1.46	3.13	4.00	3.10
Shenyang	NE	−9.30	−9.34	−9.15	−9.04	−67.77	−68.31	−66.51	−66.60	6.63	6.44	6.68	5.72
Chengde	N	−8.43	−8.25			−63.17	−62.15			4.30	3.84		
Dandong	NE	−8.81	−8.24			−60.88	−56.84			9.59	9.11		
Beijing	N	−9.61	−9.92	−9.97	−9.73	−64.65	−66.44	−66.36	−64.89	12.23	12.90	13.40	12.95
Tianjin	N	−6.91	−5.76			−54.51	−49.04			0.73	−2.94		
Tangshan	N	−7.69	−7.86	−7.89		−57.13	−58.05	−57.45		4.41	4.86	5.70	
Baoding	N	−8.68	−8.72	−8.90		−63.49	−63.82	−64.27		5.94	5.96	6.90	
Cangzhou	N	−10.43	−10.46	−10.68	−10.04	−77.24	−77.61	−78.30	−76.52	6.18	6.03	7.11	3.78
Dalian	NE	−6.77	−6.56	−7.31	−6.96	−51.18	−50.30	−53.93	−52.73	2.94	2.15	4.56	2.91
Hengshui	N	−10.88	−10.80			−80.24	−79.60			6.81	6.83		
Dongying	N	−7.20	−7.21	−6.32	−5.47	−55.13	−53.89	−50.08	−46.18	2.47	3.76	0.44	−2.45
Yantai	N	−6.05	−6.40	−6.42	−5.60	−45.97	−48.43	−46.84	−44.20	2.45	2.74	4.54	0.63
Weifang	N		−8.23	−8.34	−7.61		−60.35	−60.20	−56.68		5.50	6.49	4.19
Lhasa	QP	−17.29	−17.39	−17.29	−17.06	−130.29	−131.19	−128.67	−127.71	8.02	7.92	9.63	8.76
Gannan	QP	−9.97	−10.14	−10.57	−9.96	−67.54	−68.79	−68.87	−67.36	12.23	12.36	15.66	12.29
Dingxi	N	−10.06	−10.40	−10.58	−10.26	−69.14	−71.27	−68.59	−70.33	11.38	11.92	16.04	11.72
Longnan	N		−10.43	−10.68	−10.40		−69.21	−69.99	−68.99		14.24	15.46	14.22
Chengdu	SW	−12.22	−12.00	−12.35	−12.42	−84.80	−82.65	−83.84	−84.33	12.93	13.33	14.93	15.00
Nyingchi	QP	−13.89	−13.41	−13.91	−14.06	−99.61	−94.76	−99.37	−100.98	11.48	12.49	11.91	11.46
Xichang	SW	−8.01	−9.80	−7.42	−9.78	−68.59	−75.40	−63.77	−76.64	−4.51	2.98	−4.42	1.64
Panzhihua	SW	−14.31	−14.63	−13.49		−106.54	−108.98	−96.87		7.98	8.07	11.02	
Baoshan	SW	−9.93	−9.76		−9.73	−70.64	−68.61		−68.70	8.79	9.45		9.13
Kunming	SW	−11.11	−11.01	−11.23	−11.48	−82.66	−81.94	−81.75	−83.99	6.26	6.17	8.12	7.82
Qujing	SW	−9.60	−9.06			−72.49	−68.62			4.34	3.83		
Simao	SW	−9.02	−7.79	−8.57	−9.35	−64.87	−58.82	−60.04	−67.16	7.28	3.53	8.55	7.61
Wenshan	SW	−10.69	−9.81	−9.11	−6.39	−75.38	−69.36	−65.30	−58.98	10.16	9.10	7.57	5.20
Tianshui	N	−8.61	−8.95	−9.27	−9.16	−58.07	−60.51	−60.02	−60.33	10.79	11.12	14.15	12.94
Zhengzhou	N	−8.09	−8.83	−8.15		−57.43	−63.73	−54.73		7.30	6.94	10.48	
Kaifeng	N	−8.15	−8.07	−6.41	−8.11	−60.35	−58.76	−45.10	−60.71	4.85	5.83	6.20	4.16
Hanzhong	N	−8.91	−8.54	−8.22	−8.24	−58.95	−55.20	−52.84	−52.98	12.30	13.11	12.93	12.96
Xianyang	N	−11.58	−11.54	−11.55	−11.24	−84.51	−84.49	−83.45	−82.12	8.11	7.87	8.93	7.80
Enshi	SE	−8.11	−6.90			−54.28	−39.68			10.56	15.48		
Wuhan	SE	−8.77	−8.64	−8.01	−9.27	−59.01	−57.54	−51.04	−64.36	11.19	11.62	13.03	9.77
Chongqing	SW	−5.45	−7.35	−4.46	−7.45	−40.72	−44.71	−24.39	−50.38	9.79	14.08	11.28	9.24
Yueyang	SE	−4.62	−4.65			−29.65	−28.08			7.34	9.08		
Changsha	SE	−5.25	−5.14			−32.78	−27.77			9.21	13.36		
Bijie	SW	−9.32	−9.21	−8.72	−9.72	−63.12	−61.32	−57.43	−62.02	11.43	12.34	12.31	14.13
Zunyi	SW		−6.34				−41.09				9.59		
Tongren	SW	−7.22	−6.69	−7.77	−7.29	−44.88	−40.07	−45.68	−44.62	12.90	13.48	16.52	13.73
Huaihua	SE		−6.05	−7.02	−6.87		−38.75	−41.79	−41.69		9.61	14.40	13.24
Hong Kong	SE	−5.10	−5.06	−5.78	−5.38	−30.95	−30.58	−32.83	−31.96	9.83	9.92	13.38	11.11
Guiyang	SW	−6.14	−7.34	−8.35	−7.25	−42.53	−51.12	−52.78	−48.06	6.55	7.58	14.02	9.92
Guilin	SE	−5.82	−5.79	−6.81	−6.48	−34.73	−32.90	−39.10	−39.28	11.83	13.38	15.39	12.56
Zaozhuang	N	−6.05	−5.36	−6.36	−6.61	−46.19	−41.51	−46.72	−48.98	2.24	1.37	4.13	3.87
Xuzhou	N	−10.42	−10.36	−10.80	−10.29	−76.73	−76.07	−77.01	−76.03	6.66	6.80	9.41	6.61
Suzhou	N	−8.12	−8.32	−8.18	−7.47	−57.81	−58.11	−57.51	−55.65	7.13	8.41	7.96	4.11
Yancheng	SE	−5.15	−4.39	−5.74	−5.48	−37.83	−31.04	−39.92	−39.79	3.38	4.04	5.98	4.07
Nantong	SE	−7.61	−7.37			−51.30	−48.78			9.61	10.14		
Hefei	SE	−5.84	−5.41			−40.96	−35.47			5.79	7.78		
Ma’anshan	SE	−7.69	−6.87			−52.06	−44.77			9.50	10.21		
Shanghai	SE	−4.95	−4.22	−5.43	−4.48	−32.85	−26.68	−34.01	−30.67	6.78	7.07	9.46	5.18
Shaoxing	SE	−7.75	−7.23			−52.07	−45.09			9.95	12.78		
Hangzhou	SE		−6.40				−40.18				11.04		
Quzhou	SE	−6.97	−6.59	−6.91	−6.63	−44.79	−40.92	−41.39	−41.99	10.96	11.76	13.92	11.04
Lishui	SE	−8.24	−7.11	−7.90	−7.24	−54.74	−43.40	−50.32	−46.67	11.21	13.47	12.87	11.28
Fuzhou	SE	−6.25	−5.96	−6.60	−7.47	−40.60	−34.28	−41.74	−47.84	9.42	13.39	11.04	11.95
Longyan	SE	−6.39	−6.59	−6.55	−6.75	−37.88	−39.04	−38.89	−40.36	13.25	13.66	13.49	13.62
Liuzhou	SE	−6.14				−35.17				13.93			
Shaoguan	SE	−6.25	−5.55	−6.66	−7.09	−38.86	−32.53	−39.03	−42.28	11.15	11.88	14.28	14.46
Xiamen	SE	−6.24	−6.13	−6.64	−6.95	−39.55	−37.22	−41.80	−44.97	10.38	11.85	11.33	10.62
Bose	SW		−8.61	−9.12	−9.03		−59.88	−59.76	−61.60		9.03	13.17	10.60
Guangzhou	SE	−6.16	−5.49	−6.38	−6.53	−39.69	−31.97	−40.62	−42.75	9.58	11.96	10.44	9.51
Nanning	SE	−7.94	−7.76	−7.95	−8.54	−54.79	−54.35	−53.91	−59.61	8.70	7.69	9.68	8.71
Shenzhen	SE	−5.55	−5.03	−6.24	−6.03	−35.72	−31.55	−37.80	−38.15	8.64	8.71	12.10	10.09
Qinzhou	SE	−6.55	−6.16			−41.10	−38.79			11.32	10.49		
Zhanjiang	SE	−6.53	−4.23	−5.12	−7.02	−45.68	−28.68	−30.35	−48.74	6.58	5.16	10.62	7.42
Haikou	SE	−6.49	−6.64	−6.31	−6.63	−42.49	−44.71	−41.84	−45.14	9.46	8.42	8.60	7.92
